# Operational characteristics of an antibody detecting point of care test for *Taenia solium* infections in a community and hospital setting

**DOI:** 10.1186/s12879-021-06320-3

**Published:** 2021-06-25

**Authors:** Chishimba Mubanga, Kabemba E. Mwape, Isaac K. Phiri, Chiara Trevisan, Mwemezi Kabululu, Gideon Zulu, Inge Van Damme, Veronika Schmidt, Pierre Dorny, Sarah Gabriël

**Affiliations:** 1 Department of Clinical Studies, School of Veterinary Medicine, University of Zambia, Lusaka, Zambia; 2 Department of Veterinary Public Health and Food Safety, Faculty of Veterinary Medicine, Ghent University, Merelbeke, Belgium; 3 Department of Biomedical Sciences, Institute of Tropical Medicine, Antwerp, Belgium; 4 Tanzania Livestock Research Institute (TALIRI) – Uyole, P. O. Box 6191, Mbeya, Tanzania; 5 Provincial Health Office, Ministry of Health, Chipata, Zambia; 6 Department of Neurology, Centre for Global Health, Klinikum Rechts der Isar, Technical University Munich, Munich, Germany; 7 Centre for Global Health, Institute of Health and Society, University of Oslo, Oslo, Norway; 8 Department of Virology, Parasitology, and Immunology, Faculty of Veterinary Medicine, Ghent University, Merelbeke, Belgium

## Abstract

**Background:**

Diagnostic test evaluation includes measures of performance and assessment of operational characteristics. The latter focuses on end-user understanding of instructions to perform the test, ease of use, test turnaround time and ease of result interpretation. This study aimed to assess user comprehension of training for and ease of use of a *Taenia solium* point of care test (TS POC) evaluated in a community and hospital setting in Zambia and Tanzania, respectively.

**Methods:**

The TS POC is a three-step in-house-produced rapid diagnostic test (RDT) for the simultaneous detection of taeniosis (TST) and cysticercosis (TSCC) antibodies. Data collected by administering questionnaires to 29 end-users and from the main evaluation database was analyzed quantitatively.

**Results:**

End-users (28/29, 97%) perceived that the training they received for performing the test was sufficient. They performed 4080 tests, of which 80 were invalid. The community-based study and TST tests had higher invalid rates. The overall result interpretation was within the acceptable range of RDTs with an overall disagreement between readers of 3.3%. The Kappa coefficient of agreement was 85 and 82% for TSCC and TST, respectively. There was more disagreement among readers in the community-based study.

**Conclusion:**

End-users rated the TS POC kit moderate in terms of ease of use citing long test turnaround time and difficulties in using the blood transfer device. Overall, the operational performance of the TS POC kit and end-users was within the established acceptable performance range.

**Supplementary Information:**

The online version contains supplementary material available at 10.1186/s12879-021-06320-3.

## Introduction

Diagnostic test evaluation includes measures of performance and assessments of operational characteristics. Performance characteristics incorporate measures of diagnostic accuracy while operational characteristics include end-user practices in terms of understanding of test operational instructions, test robustness under different storage conditions, simplicity or ease of use, test user acceptability, result ease of interpretation, and turnaround time [1]. Operational characteristics are usually qualitative and subjective.

Operational characteristics of diagnostic tests may have a bearing on performance characteristics. End-user performance has been reported to affect diagnostic accuracy, often as end-user errors [2–4]. Studies have demonstrated end-user errors in the use of rapid diagnostic tests to include broadly, not understanding or not adhering to instructions relating to safety or maintaining the quality of diagnostic tests. Errors have also been reported in blood sampling and dispensation as well as the use of buffer and reading of results [2]. Therefore, several studies evaluating operational characteristics have included assessments of the clarity of instructions for use [3, 5] and ease of use or test performance simplicity [6–8]. These parameters provide insight in explaining the performance outcomes of diagnostic evaluations and identify points of improvement.

While often operational characteristic evaluations are conducted upon commercialization of a test, and in comparison with other similar tests for a particular target condition [3, 6–11], we contend that this assessment needs to be conducted for tests under development as well, to aid in the clarification of performance deficiencies and to inform on potential weaknesses/problems for the next stage of test kit development. The *Taenia solium* diagnostic project (SOLID) evaluated a new rapid antibody detecting point of care test (TS POC) for the surveillance of *T. solium* infections in humans. The evaluation was conducted in two endemic countries, Zambia and Tanzania. The setting in Zambia was community-based while the setting in Tanzania was hospital-based. The TS POC is a prototype standard lateral flow assay that simultaneously detects antibodies against taeniosis and cysticercosis, caused by the adult and larval stages of *T. solium*, respectively. The assessment of the operational characteristics was undertaken within the framework of the performance evaluation.

This study aimed to concurrently get feedback on user training and ease of use of the TS POC among end-users in Zambia and Tanzania during the community and hospital-based evaluations. We assessed perceptions about training clarity and complexity of the test technique, the time needed to perform the test, time to results, ease of result interpretation, and general ease of use of the test [9]. Finally, we compared these with variables obtained from the main test evaluation database such as levels of reader disagreement and test invalid rates. We hypothesized that the performance of TS POC kit and end-users were within the established, acceptable operational performance range of similar tests.

## Materials and methods

Trial registration: The study was registered in the Pan African Clinical Trial Registry PACTR201712002788898 on 5th December, 2017.

### Setting

The study was carried out in two settings: a community setting (Sinda district) in a rural area of Zambia (*n* = 14 end users), where nine professional health workers and five community health workers were testing people using the TS POC; and a hospital-based setting in Mbeya (Ifisi, Tukuyu) and Songwe (Vwawa) regions, Tanzania, involving three hospitals (Ifisi, Tukuyu, and Vwawa) where 19 nurses were involved in performing the test. The study sites were selected because of low sanitation levels, presence of free range pigs and known, *T. solium* endemicity. In Zambia, end-users tested participants during recruitment in the continuous presence of a scientist while in Tanzania, nurses were working independently and were only observed recruiting participants during follow up sessions. Each group had a step by step pictorial (job aid card (see Additional file 2: Annex 2)A) instructions on how to perform the test.

### End-users

In total, 33 test end-users were involved in the recruitment of SOLID study participants/patients. The 33 end-users were health professionals and community health workers (CHW). The health professionals had formal college education. The CHW had secondary education. Years of experience varied among end users, however all had previous experience in performing rapid tests for malaria and for some also HIV RDT. Test end-users were recruited for the SOLID study because they were either members of staff or neighborhood health committees working in health facilities selected for the study. Fourteen (2 clinicians, 3 nurses, 1 environmental health technician (EHT), 5 CHW and 3 laboratory technicians) and 19 (all nurses) were originating from Zambia and Tanzania, respectively. Of the 33 end-users, only 29 responded to the questionnaire, 13 from Zambia and 16 from Tanzania. Four nurses, one from Zambia and three from Tanzania were not available at the time. Among the respondents, there were 12 women and 17 men. The age range was 22 to 59 years with a mean age of 39 (1st quartile 32, 3rd quartile 44).

### The *Taenia solium* point of care test (TS POC)

**Fig. 1:**
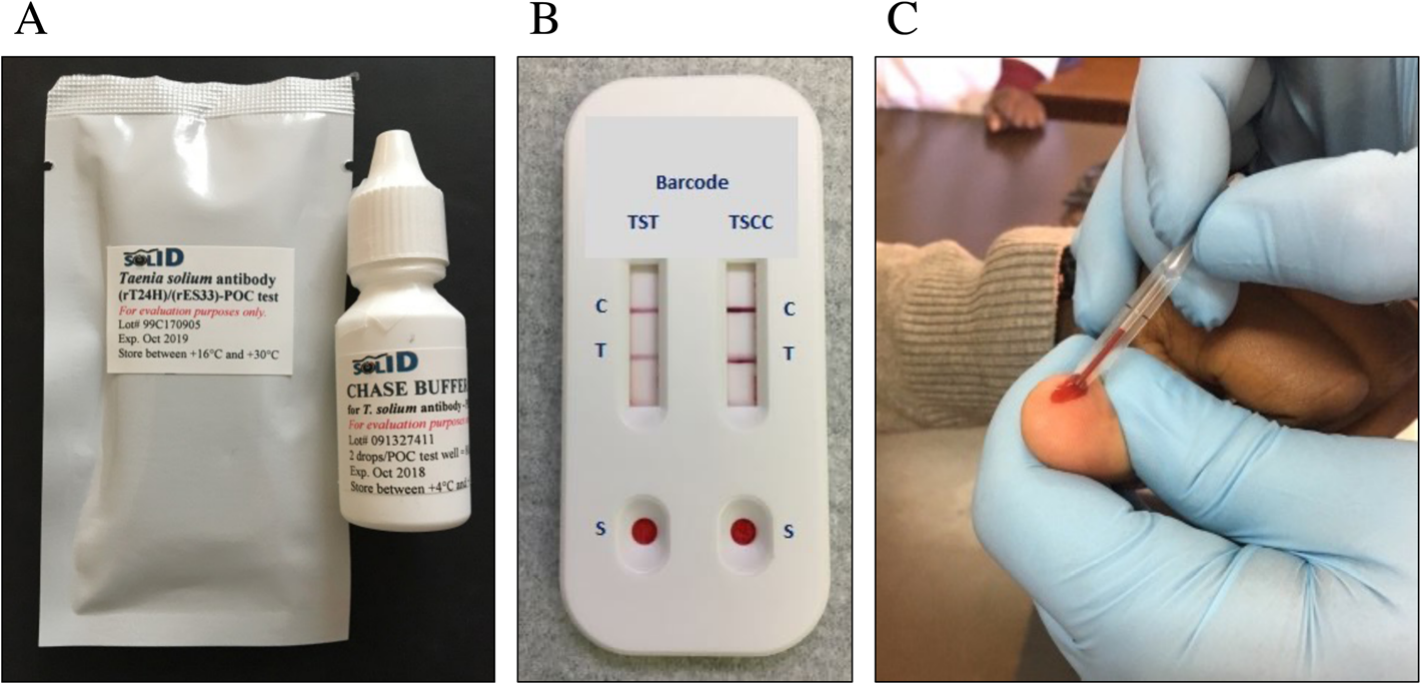
The Taenia solium point of care test kit (TS POC). **A** TS POC kit and chase buffer in a dropper bottle; **B** The TS POC cassette; **C** Collecting blood with the squeezable calibrated micropipette

The TS POC (Fig. 1B below) is an in-house prototype rapid diagnostic test for the simultaneous detection of taeniosis (TST) and cysticercosis (TSCC) antibodies in a lateral-flow format based on two recombinant proteins, rES33 and rT24H, respectively [12, 13]. The test kits (each comprising of one TS POC cassette and one desiccant sealed together in an aluminum pouch) were supplied together with chase buffer in dispensing dropper bottles (Fig. 1A) and additional calibrated squeezable micropipettes (Microsafe Capillary Tubes 20ul, SafeTec, PA, USA) (Fig. 1C) by the Klinikum Rechts der Isar, Technical University Munich, Germany, and the Division of Parasitic Diseases and Malaria, Center for Global Health, Center for Disease Control and Prevention (CDC), Atlanta, Georgia, USA, and shipped from the latter to the study sites. On the TS POC cassette, each test strip has a sample port (S), a test line (T) and a control line (C) (Fig. 1B). The rest of the materials needed (such as lancets, swabs with alcohol) were locally purchased. The preliminary TS POC laboratory performance was estimated as follows; for cysticercosis a sensitivity of 88–93% and specificity of 99%; for taeniosis a sensitivity of 82%, and a specificity of 99% (CDC, TUM, unpublished). The manuscripts for the performance evaluation (diagnostic accuracy) are in preparation.

In summary, the test procedure involved cleaning the finger with an alcohol swab and pricking the finger side with a lancet. Blood was collected with a micropipette and dispensed onto one sample port of the cassette and then to the other port using a separate micropipette. Immediately afterwards, two drops of chase buffer were applied to each sample port and the results were read 20 min after the commencement of the flow (Detailed procedure on the job aid card in Additional file 2: Annex 2). The results were read independently by two end-users, with the test performer reading as second. In case of disagreement or uncertainty, a third person (a scientist in Zambia, and a third nurse in Tanzania) read the results. In that case, the results recorded were based on the reading of the third reader. A test result was considered valid if the control lines appeared. In case of an invalid test, the procedure was repeated only once.

### End-user training

End-users underwent theoretical and practical training in *T. solium* infections and the use of the TS POC kit as well as interpretation of test results. The theory included the life cycle of the tapeworm, the TS POC design, operating the test, result reading and interpretation, invalid results and trouble shooting as well as transport and storage requirements of the kit. The practical part included an initial demonstration of how the test operates followed by a session where each end user could practice the test. In both Zambia and Tanzania, end-users practiced performing the test during the training until they were sufficiently familiar with the procedures. We assessed the end-users through observations, interactions, and feedback. An end-user was deemed sufficiently trained if he/she attended and participated in all sessions and demonstrated proficiency in performing the test and interpreting the results on the job aid card.

In Zambia, the first training was a half-day training involving all health staff and CHW from all neighborhood health committees. Subsequently, each recruitment mission began with refresher trainings (7 trainings) involving all health staff and only those CHW in whose area the recruitment was being done. In Tanzania, for each participating hospital (Ifisi, Tukuyu, and Vwawa) there was one, two-day training organized for the nurses. The difference between the Zambia and Tanzania training was that the Tanzania training included data capturing using tablets. Subsequent supportive training and follow up sessions were given to support staff where deviations from standard operating procedures and mistakes were observed. In both countries, we delivered training through short interactive presentations by the project medical doctors and scientists. Training was conducted in English with translations into Chewa and Swahili for Zambia and Tanzania, respectively.

### Data collection

A questionnaire (Additional file 2: Annex 1) was designed and pretested to health workers and community health workers who were not part of the study. The questions in the questionnaire were designed to get feedback on training, the technical complexity of the use of the test kit, the time needed to perform the test, time to results and ease of result interpretation. The questionnaire was then converted to EpiCollect 5 (Centre for Genomic Pathogen Surveillance, Wellcome Sanger Institute, Cambridge shire, UK) to allow electronic data capture. The questionnaire was in English and was administered by one researcher (CM) in Zambia and another (MK) in Tanzania. The researcher translated the questions to the end-users who were more comfortable with the local language (Chewa or Swahili). The questionnaire had 30 questions in total, 26 closed and 4 open-ended questions.

Data such as, the number of tests performed by each end-user, invalid tests and data for reader agreement was collected from the SOLID main evaluation database as of 8th October 2019.

The questionnaires were administered between June and September 2019.

### Data analysis

Quantitative data from the questionnaire was presented as proportions [4, 9]. A thematic analysis of answers to open-ended questions was done to assess the views of the TS POC kit end-users about its positive and negative sides as well as their recommendations for improvement [14]. The frequency of recurring themes was evaluated and used to order themes in descending or ascending frequency. Further, we analyzed information from the main evaluation database to get numbers/proportions of tests performed and invalid tests as well as to calculate reader agreement. Cohen’s Kappa statistic was used to determine the level of reader agreement as part of result interpretation assessment.

## Results

### Training

In total 50% of the end-users had attended 4 or more trainings (Table 1).

**Table 1:** Training attendance by end-users

	Number of trainings attended			
	1	2	3	≥4
Zambia (overall)	1	1	2	9
Clinical officers	1	0	0	1
Community health workers	0	1	2	3
Environmental Health Technician	0	0	0	1
Laboratory technicians	0	0	0	2
Nurses	0	0	0	2
Tanzania				
Nurses	1	4	5	6
Total	2	5	7	15

The majority of end-users (28/29) reported that the training was sufficient.

### Frequency of TS POC test use

**Fig. 2:**
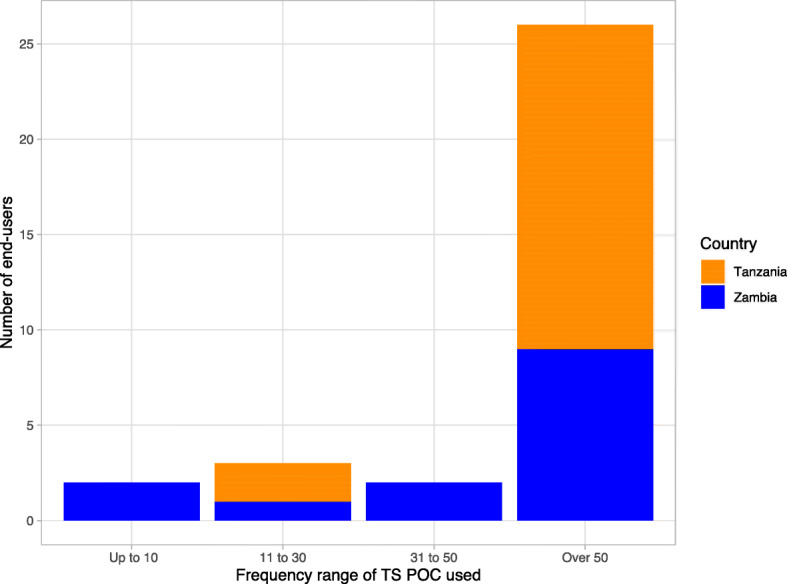
Frequency of Taenia solium point of care test used by all (33) end-users

The majority of end-users (27/33) from the main evaluation database performed more than 50 TS POC tests (Fig. 2) (Detailed summary data are shown in Additional file 3: Table S2).

### Experience with the blood transfer device (the squeezable calibrated micropipette) and application of chase buffer

#### Experience with the blood transfer device

Overall, 17/29 of end-users found the use of the squeezable micropipette easy while 12/29 found it very easy. However, during blood collection using the micropipette, 19/29 end-users reported sometimes collecting insufficient blood, 9/29 never experienced this and 1 end-user always experienced this. Furthermore, 12/29 end-users reported sometimes having a bubble of air in the pipette, 16/29 never experienced it and 1 end-user always experienced it. In terms of discharging all the blood from the micropipette, 13/29 of the participants reported sometimes experiencing difficulties, 1/29 always experienced difficulties while 15/29 had no difficulties.

#### Application of chase buffer

All end users found it easy or very easy to apply the chase buffer. In terms of applied volume, 8/29 reported sometimes having put more than 2 drops.

### Perception of time for performing the test, waiting and start of the flow

#### Time for performing the test

This was defined as the time spent on setting up TS POC on the table, putting on gloves, cleaning up the finger, collecting blood, transferring the blood to the TS POC sample ports and applying the chase buffer; where 18/29 end-users reported performing the test in 3–5 min, 7/29 reported less than 2 min, 4/29 reported more than 5 min.

#### Start of the flow

The average time to start of the flow was estimated at less than 2 min for both TST and TSCC by 19/29 of end-users and between 3 and 5 min by 7/29 end-users for TSCC and 6/29 for TST, respectively. It was estimated at 6–10 min by 2/29 end-users for TSCC and 3/29 end-users for TST. When asked if there was a difference in the start of the flow between TST and TSCC, 16/29 end-users responded that sometimes there was a difference, 12/29 said the difference was always there while 1/29 was not sure. When asked whether the difference in the start of the flow reported by 12/29 end-users had a pattern, 11/29 said TSCC started running first, while the other 11/29 said TST ran first, 6/29 did not observe any pattern and 1/29 was not sure.

### Test interpretation

#### Visibility of control lines

Test validity was defined by the presence of the control line. In total, 25/29 end-users reported that it was easy to see the control lines and 4/29 reported that it was very easy. Overall, 4080 tests were performed by the end-users, 1254 in the community setting (Zambia) and 2826 in the hospital setting (Tanzania). Overall, 80 tests were recorded not to have a visible control line on either TST, TSCC or both (Additional file 3: Table S2 disaggregated data by country and each end-user).

#### Invalid tests

Invalid tests (lack of the control line(s)) were always related to incomplete or no flow. Overall, 80 of the TS POCs were invalid on either the TSCC, TST or both. By country, Zambia reported 57 of the 80 invalids while Tanzania reported the remaining 23 invalid tests (Additional file 3: Table S2). In both countries, more invalids occurred on the TST (59/80, 74%). Overall, 23/33 end-users recorded an invalid test when performing the TS POC. At individual end-user level (Additional file 3: Table S2), six end-users had an invalid rate over 5%: 15.0% (5/34) Clinician, Zambia; 9.0% (2/22) Nurse, Tanzania; 7.0% (10/142) Clinician, Zambia; 7.0% (24/350) EHT, Zambia; 6.0% (1/16) Nurse, Tanzania; and 6.0% (1/14) CHW, Zambia.

### Observation of a positive test

In total 28/29 of end users reported having observed a TST+ result while 1/29 did not. All end users reported having observed a TSCC+ result.

The responses about the visibility of the test lines seemed similar for TST and TSCC; very easy (TST 8/29, TSCC 10/29), easy (TST 20/29, TSCC 18/29) and difficult 1/29 for both. The occasional use of a torch to assess the presence of the test lines was reported by 25/29 of the end-users. Nevertheless, when questioned about having doubts when reading the test, 18/29 end-users reported this to happen in rare cases, and 11/29 sometimes. The responses were compared with the provisions of “unclear strip” and “not being sure” about the results provided during data capture when performing the test. From the main evaluation database, the “unclear” result has been recorded 3/4080 (0.07%) times and only in Tanzania. For the provision of not being sure, a doubting result (TST? and/or TSCC?) was recorded overall 225 (Zambia 7, Tanzania 218) times by the end-users. Of these times, 214/225 (95.0%) indicated doubt on the TST results (TST?) while 11/225 (5.0%) indicated doubt on the TSCC (TSCC?) results. The doubting results were recorded by first reader 122/225 (54.0%), second reader 96/225 (43.0%) and third 7/225 (3.0%). The 7/225 then became indeterminate results (5/4080 TST, 2/4080 TSCC).

From the main evaluation database, reader agreement and disagreement were analyzed. We evaluated the reader agreement between the two readers by calculating Cohen’s Kappa coefficient of agreement. The calculated coefficient for TSCC of 85% (57–99%; Zambia 65% (57–71%), Tanzania 95% (90–99%) is termed as almost perfect agreement. Kappa coefficient for TST was 82% (16–100%; Zambia 47% (16–78%), Tanzania 100% (95–100%)), also almost perfect agreement. Overall end-users disagreed in 3.3% (136/4080) about the TS POC results. The disagreement was higher from community studies in Zambia at 8.6% (108/1254) compared to the hospital setting in Tanzania at 0.1% (28/2826). The disagreement was 0.2% (8/4080) and 3.1% (128/4080) on TST and TSCC respectively.

### Previous experience in POC use and ranking of the TS POC prototype

We asked end-users to list up to 5 commercial rapid diagnostic tests they had used before, beginning with the most frequently used. The results are as follows;

1. HIV (17/29), Malaria (12/29)
2. HIV (20/29), Malaria (4/29), Syphilis (5/29),
3. HIV (16/29), Pregnancy (4/29), Malaria (4/29), Syphilis (5/29)
4. Blood glucose (18/29), Pregnancy (5/29), Hepatitis B (1/29), Syphilis (3/29), and HIV (2/29)
5. Pregnancy (26/29), Hepatitis B (1/29), HIV (1/29), Syphilis (1/29)

**Fig. 3:**
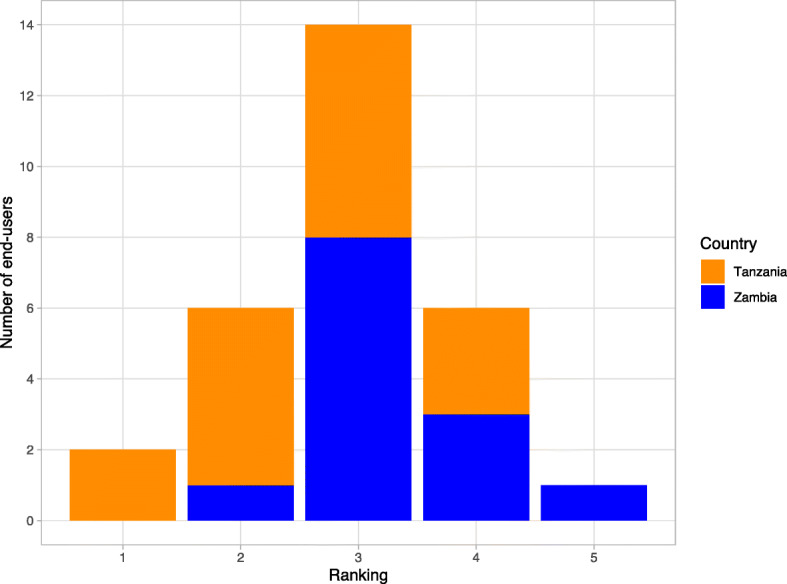
Ranking of the TS POC kit by end-users

We then asked them to rank the TS POC kit against these tests they had used before in terms of ease of use (1 Easiest, 5 Most Difficult). The results are as shown in Fig. 3.

The majority of end-users (14/29) ranked the TS POC kit third in terms of ease of use, while 7/29 ranked it second and 6/29 ranked it 4th.

The reasons reported for ranking other tests higher (easier) than the TS POC in order of reducing frequency were as follows: (1) Less time to wait for results (2) Smaller/any amount of blood for testing (3) Taking blood only once (4) Blood transfer is easier (5) Flow is more rapid (6) Easier to read and use (7) Not so many procedures to follow (8) Test for one disease condition (9) Buffer can be stored at room temperature.

### Positive aspects of the TS POC kit

Each end-user was asked to give up to 3 positive aspects of the kit according to their experience. The majority felt that it was easy to use (41%) and could diagnose two diseases simultaneously (19%) (Additional file 1: Table S1).

### Challenges encountered with using the TS POC kit

The main challenges end-users encountered using the TS POC kit were: delayed flow (26%), long waiting time for results (26%), difficulties in micropipette use (23%) and “unclear” results (11%) (Additional file 1: Table S2).

### End-user recommendations for TS POC kit improvement

The main recommendations were: shorten the time to wait for results (25%) and improvement on overall quality and buffer not requiring refrigeration (25%) (Additional file 1: Table S3).

## Discussion

Based on the results of the study, the respondent end-users showed that the majority of them felt they had received sufficient training. Most end-users performed more than 50 TS POC tests, which provided them sufficient experience with the TS POC kit on which to give feedback. The participant’s feedback on the supplied micropipette was generally that it was easy to use. However, two-thirds reported sometimes not managing to draw blood up to the mark and others experienced sometimes bubbles in the pipette. Correspondingly, the micropipette was also reported as the third challenge as well as the third recommendation on the list of improvements to the TS POC. Difficulties in blood collection and transfer leading to inadequate volume is a problem well documented in RDT use [4, 6, 15–17]. Insufficient blood may lead to false negatives, while too much blood may cause the “prozone” effect and reduce strip visibility [2]. We observed the end-users from the community set up struggled the first day but progressively improved over time (Personal observation, CM). This is similar to what another longitudinal study observed, a marginal increase in blood collection performance [17]. Perhaps increasing the practical part of the training as well as changing the transfer device could reduce blood transfer difficulties. The blood transfer stage may affect the outcome of diagnostic performance though the magnitude could not be estimated.

Diagnostic turnaround time for RDTs included time to perform the test, time for the flow to start and the mandatory waiting time for the test to run [18], 20 min in this case. From the results, time to sample flow and the waiting time before the results were major issues raised. Indeed, they increased the overall turnaround time and subsequently reduced the throughput. Turnaround time has been reported as a third factor behind the sensitivity and cost upon which professionals base their choice of diagnostic tests. Another study reported that 15 min is acceptable to most professionals [19]. Nevertheless, the current 20 min waiting time on average is within the 30 min threshold in the target product profile for taeniosis and cysticercosis [20]. The overall impact of the differing time to flows between TST and TSCC in this study was mainly an increased turnaround time. In some cases, the delayed flow led to invalid tests when the flow did not reach the control line.

The higher rate of invalid tests for TST (1.4%, 59/4080) was lower than the threshold set for HIV and malaria rapid tests of not more than 5% [7, 11, 21]. The invalid rates were higher from the community setting than from the hospital setting. This could be due to the open-air testing, during which the cassette and blood sample were exposed to weather elements. This could be compounded by end-user errors such as delay in the application of buffer leading to blood drying. A timeline analysis showed that the invalid results occurred randomly throughout the entire period of testing (results not shown). Nearly two-thirds of the end-users had at least one test invalid. The four end-users with higher invalid rates had performed only a few tests. The higher rate of invalid tests for TST was observed across both countries. The probable reason for this was the differences in pressure points on the cassettes as these were manually assembled.

Overall, end-users reported that the test lines were easy to see despite having had to sometimes use a torch to clearly see. This seems supported by the low number of doubting results. The doubting result was also noticeably higher from the hospital setting than from the community setting. This could be a reflection of the lack of immediate back-up from the scientists who were only occasionally present in Tanzania during supervisory visits.

The Kappa coefficient of reader agreement was calculated at 85 and 82% for TSCC and TST respectively, an almost perfect agreement, which is within the recommended minimum of 80% in health research [22]. However, both the Kappa coefficient and percentage disagreement from the community setting in Zambia showed higher disagreement among readers compared to the hospital setting in Tanzania. The possible explanation for this is that the primary readers of the test in the community setting were the CHWs who had generally lower training, knowledge, and infrequent RDT experience compared to nurses in the hospital setting. Reader disagreements could also be due to subjective interpretation of [23] and ignoring faint test lines, considering them as negatives which may underestimate positivity [2, 17, 24] and increase discordant readings.

The moderate ranking of the TS POC in the context of RDT experience was largely based on turnaround time, the volume of and the number of times of collecting blood. The TS POC prototype is, in fact, two tests in one cassette hence the necessity for more blood drops. Re-designing the TS POC by putting two test lines for TST and TSCC on one strip with a single control line similar to the SD BIOLINE MALARIA AG P.F/PAN test (Abbott diagnostics) would already deal with some of the reasons for the ranking.

This study relied on perceptive feedback whose limitations are well known. The number of tests performed by an individual was not standardized, therefore, end-users had a varied experience. End-users who performed very few tests like the ones who performed 2 or 6 tests had limited experience on which to give feedback. Nevertheless, even these “first” impressions were taken on board [24]. Sub-analysis of variables like profession was not done because the numbers making up these and other subgroups were very few to give statistically meaningful conclusions. However, disaggregated data is presented in Additional file 3: Table S2. Despite these limitations, the study brings out valuable information important for diagnostic tests under development.

## Conclusion

Our study found that the end-users perceived they were sufficiently trained for TS POC evaluation. The test technique was relatively easy given the number of valid tests performed. The performance of the TS POC kit and end-users based on invalid tests and reader agreement, respectively was within the acceptable operational performance range. The TS POC device has been ranked moderately easy to use with the main drawbacks reported being a long turnaround time and the less easy to use blood transfer device. Re-designing the TS POC to a dual antigen each with a test line but single strip and using a more acceptable blood transfer device would deal with the issues raised by the end-users.

## Supplementary Information

**Additional file 1: Table S1.** Positive aspects of the TS POC according to end-users. **Table S2.** Challenges end users encountered when using the TS POC Test. **Table S3.** End-user recommendations for TS POC improvement.

**Additional file 2: Annex1.** Questionnaire. **Annex 2.** *T. solium* user manual (job aid card).

**Additional file 3: Table S1.** Training attendance by end-users. **Table S2.** Invalid test rates by an individual end-user.

## Data Availability

The data set used in this manuscript is available from the corresponding author on reasonable request.

## Abbreviations

CDC: Centers for Disease control and prevention
CHW: Community health worker
EC: Ethical clearance
EHT: Environmental Health Technician
HIV: Human immunodeficiency virus
RDT: Rapid diagnostic test
SOLID: Taenia solium diagnostic project
TSCC: Taenia solium cysticercosis
TS POC: Taenia solium point of care test
TST: Taenia solium taeniosis
TUM: Technical university of Munich
