# Supplementary material for: Operational characteristics of an antibody detecting point of care test for Taenia solium infections in a community and hospital setting
Source: BMC Infect Dis. 2021 Jun 25;21:607. doi: 10.1186/s12879-021-06320-3 (PMC8235832; doi:10.1186/s12879-021-06320-3)
Supplement: Supplementary file 1 — Additional file 1: Table S1. Positive aspects of the TS POC according to end-users. Table S2. Challenges end users encountered when using the TS POC Test. Table S3. End-user recommendations for TS POC improvement. [file 12879_2021_6320_MOESM1_ESM.docx]

*Supplementary tables*

*Table 1 Positive aspects of the TS POC according to end-users*

| Rank | Reason (theme) | Recurring frequency % |
| --- | --- | --- |
| 1 | Easy to use | 41 |
| 2 | Diagnose two diseases | 19 |
| 3 | Provide correct results “and exclude false beliefs on epileptic cases” | 15 |
| 4 | Quick results - “Results obtained without waiting for long” | 10 |
| 5 | Clarity of results - provides “easy to read results” | 8 |
| 7 | “Less blood is needed compared to other tests” | 3 |
| 8 | “Equipment was readily available” | 3 |
| 9 | “Easy storage” | 2 |

*Table 2 Challenges end users encountered when using the TS POC Test*

| Rank | Reason (theme) | Recurring frequency % |
| --- | --- | --- |
| 1 | Delayed flow | 26 |
| 2 | Long waiting time for the results | 26 |
| 3 | Difficulty in micropipette use | 23 |
| 4 | Clarity of results - “Sometimes results are unclear” | 11 |
| 5 | The requirement for a cold chain | 8 |
| 6 | Bubbles in sample port after adding the buffer | 2 |
| 7 | Invalid results when cold, “When it’s cold, it gives invalid results” | 2 |
| 8 | “Some TS POC test batches were not performing well” | 2 |

*Table 3 End-user recommendations for TS POC improvement*

| Rank | Recommendation (theme) | Recurring frequency % |
| --- | --- | --- |
| 1 | “Time to wait for results should be shortened” | 25 |
| 2 | Improve test sensitivity, quality of certain batches, the buffer should not require refrigeration | 25 |
| 3 | Micropipette - “Come up with a new micropipette to make it easy to collect blood to the required volume and which will make it easier to dispense the sample on the test” | 15 |
| 4 | Sample flow - “Make sure the sample start flowing at the same time and reduce invalids” | 10 |
| 5 | Improve on the visibility of test lines | 10 |
| 6 | More training is needed | 10 |
| 7 | Reduce the blood required for the test | 5 |
