# Supplementary material for: Operational characteristics of an antibody detecting point of care test for Taenia solium infections in a community and hospital setting
Source: BMC Infect Dis. 2021 Jun 25;21:607. doi: 10.1186/s12879-021-06320-3 (PMC8235832; doi:10.1186/s12879-021-06320-3)
Supplement: Supplementary file 2 — Additional file 2: Annex1. Questionnaire. Annex 2. T. solium user manual (job aid card). [file 12879_2021_6320_MOESM2_ESM.docx]

Operational characteristics of an antibody detecting point of care test for *Taenia solium* infections in a community and hospital setting

Chishimba Mubanga^1,2*^, Kabemba E. Mwape^1^, Isaac K. Phiri^1^, Chiara Trevisan^3^, Mwemezi Kabululu^4^, Gideon Zulu^5^, Inge Van Damme^2^, Veronika Schmidt^6, 7^, Pierre Dorny^3,8^ and Sarah Gabriël^2^ on behalf of the SOLID Consortium

1. Department of Clinical Studies, School of Veterinary Medicine, University of Zambia, Lusaka, Zambia
2. Department of Veterinary Public Health and Food Safety, Faculty of Veterinary Medicine, Ghent University, Merelbeke, Belgium
3. Department of Biomedical Sciences, Institute of Tropical Medicine, Antwerp, Belgium
4. Tanzania Livestock Research Institute (TALIRI) - Uyole, P. O. Box 6191, Mbeya, Tanzania
5. Provincial Health Office, Ministry of Health, Chipata, Zambia
6. Centre for Global Health, Department of Neurology, Klinikum Rechts der Isar, Technical University Munich, Munich, Germany
7. Centre for Global Health, Institute of Health and Society, University of Oslo, Oslo, Norway
8. Department of Virology, Parasitology, and Immunology, Faculty of Veterinary Medicine, Ghent University, Merelbeke, Belgium

*Corresponding author: [chishimbamubanga@yahoo.com](mailto:chishimbamubanga@yahoo.com), chishimba.mubanga@ugent.be

Annexes

Annex 1 Questionnaire

Questionnaire on operational characteristics of the *Taenia solium* point of care (POC) test

Introduction: We would like to ask you to answer this short questionnaire as you have been involved in using the POC test being evaluated under the SOLID project. The purpose of this questionnaire is to evaluate user comprehension of training, ease of use of the POC test and its acceptability among health care personnel who frequently use similar tests. Please take 15 minutes to answer the questions below. Be assured that your responses are for research purposes only and your anonymity is guaranteed.

Participant information

Sex: F M

Age: ………………………………..

Class of health care personnel:

Medical Doctor

Medical Licentiate/ Assistant Medical officer

Clinical Officer

Laboratory Technician

Nurse

Environmental Health Technician

Community-based Volunteer,

Other (Specify)…………………………………………………..

Please circle or fill in the answer you feel best describes your experience

1. Did you receive training in performing the *Taenia solium* POC test?

a) Yes

b) No

2. How many trainings in performing the *Taenia solium* POC tests did you receive?

a) 1 time

b) 2 times

c) 3 times

d) 4 times or more

3. Was the training on performing the *Taenia solium* POC test sufficient for you?

a) Yes

b) No

4. How many *Taenia solium* POC tests did you perform during this study?

a) Less than 10

b) Between 10 and 30

c) Between 31 and 50

d) More than 50

5. Overall, performing the *T. solium* POC test was…………………(choose from the list below)

a) Very easy

b) Easy

c) Difficult

d) Very difficult

6. The use of the micro pipettes was …………….(choose from the list below)

a) Very easy

b) Easy

c) Difficult

d) Very difficult

7. Did you experience times where blood did not get to the mark in the micro pipette?

a) No, never

b) Yes, sometimes

c) Yes, very often

d) Yes, always

8. Did you experience difficulties in discharging all the blood out of the micro pipette?

a) No, never

b) Yes, sometimes

c) Yes, very often

d) Yes, always

9. Did you experience an instance where there was a bubble or break between the blood column in the micro pipette?

a) No, never

b) Yes, sometimes

c) Yes, very often

d) Yes, always

10. Applying the chase buffer to the cassette was ………………..(choose from the list below)

a) Very easy

b) Easy

c) Difficult

d) Very difficult

11. Did you experience incidences were more drops of chase buffer were applied to the sample?

a) No, never

b) Yes, sometimes

c) Yes, very often

d) Yes, always

12. Roughly, how long did it take you to perform the test (without waiting for results, from cassette setup to putting chase buffer)

a) Less than 2 minutes

b) Between 3 and 5 minutes

c) More than 5 minutes

13. How long did you usually have to wait between the time you put the chase buffer on the sample and the beginning of the flow for TST

a) Less than 2 minutes

b) Between 3 and 5 minutes

c) Between 6 and 10 minutes

d) Between 11 and 20 minutes

14. How long did you usually have to wait between the time you put the chase buffer on the sample and the beginning of the flow for TSCC

e) Less than 2 minutes

f) Between 3 and 5 minutes

g) Between 6 and 10 minutes

h) Between 11 and 20 minutes

15. Did you ever come across a *T. solium* T positive?

a) Yes

b) No

16. Reading the TST line for taeniosis was usually………………………(choose from the list below)

a) Very easy

b) Easy

c) Difficult

d) Very difficult

17. Did you ever come across a *T. solium* TSCC positive?

a) Yes

b) No

18. Reading the TSCC test line for cysticercosis was usually …………………..(choose from the list below)

a) Very easy

b) Easy

c) Difficult

d) Very difficult

19. Reading the control lines was usually……………………..(choose from the list below)

a) Very easy

b) Easy

c) Difficult

d) Very difficult

20. Was there an instance when you had to use a flashlight to clearly see the results?

e) No, never

f) Yes, sometimes

g) Yes, very often

h) Yes, always

21. Was there an instance when where you had doubts about the results you were seeing?

a) No, never

b) Yes, but rarely

c) Yes, sometimes

d) Yes, always

22. Did you observe a difference in the start of sample flow between the TST and TSCC test lines?

a) Yes, sometimes

b) Yes, always

c) No, never

23. Was there a pattern to the start of the sample flow between the TST and TSCC test lines?

a) No, I didn’t observe any pattern

b) Yes, the TST often started running first

c) Yes, the TSCC often started running first

d) I am not sure

24. Before this *T. solium* POC test, have you ever used POC tests (Rapid diagnostic tests (RDT)) for other diseases?

a) No, never

b) Yes, rarely

c) Yes, often

d) Yes, very often

25. List up to 5 any POC tests you have used before, beginning with the most and ending with the least frequently used (e.g. Malaria RDT test)

a)………………………..

b)……………………….

c)……………………….

d)……………………….

e)……………………….

26. In terms of ease of use, how would you rank the *T. solium* POC test compared to the other tests you have listed above (1=Easiest, 5=Most difficult)

a) No. 1

b) No. 2

c) No. 3

d) No. 4

e) No. 5

f) No. 6

27. If the T. solium POC is ranked lower than any of the other tests, what features do these tests have which make them easier to use than the *T. solium* POC?

………………………………………………………………………………………………………………………………………………………………………………………………………………………………………………………………………………………………………………………………………………………………………………………………

28. List 3 positive aspects of the *T. solium* POC test you experienced

………………………………………………………………………………………………………………………………………………………………………………………………………………………………………………………………………………………………

29. List 3 challenges you encountered in using the *T. solium* POC tests

………………………………………………………………………………………………………………………………………………………………………………………………………………………………………………………………………………………………

30. What recommendations would you give to improve the *T. solium* POC test?

………………………………………………………………………………………………………………………………………………………………………………………………

Thank you for participating

Annex 2 *T. solium* user manual (job aid card)
