# Supplementary material for: Operational characteristics of an antibody detecting point of care test for Taenia solium infections in a community and hospital setting
Source: BMC Infect Dis. 2021 Jun 25;21:607. doi: 10.1186/s12879-021-06320-3 (PMC8235832; doi:10.1186/s12879-021-06320-3)
Supplement: Supplementary file 3 — Additional file 3: Table S1. Training attendance by end-users. Table S2. Invalid test rates by an individual end-user. [file 12879_2021_6320_MOESM3_ESM.docx]

Appendices

Table 1 Training attendance by end-users

|  | Number of trainings attended | | | |
| --- | --- | --- | --- | --- |
|  | 1 | 2 | 3 | ≥4 |
| Zambia (overall) | 1 | 1 | 2 | 9 |
| - Clinical officers | 1 | 0 | 0 | 1 |
| - Community health workers | 0 | 1 | 2 | 3 |
| - Environmental Health Technician | 0 | 0 | 0 | 1 |
| - Laboratory technicians | 0 | 0 | 0 | 2 |
| - Nurses | 0 | 0 | 0 | 2 |
| Tanzania |  |  |  |  |
| - Nurses | 1 | 4 | 5 | 6 |
| Total | 2 | 5 | 7 | 15 |

Appendices

*Table 2 Invalid test rates by an individual end-user*

| Country | Profession | Both control lines visible | No control line visible | Only TSCC control line visible | Only TST control line visible | Total invalid tests | Total TS POC cassettes utilized |
| --- | --- | --- | --- | --- | --- | --- | --- |
| Zambia | Clinician | 132 | 0 | 5 | 5 | 10 | 142 |
| Zambia | Clinician | 29 | 0 | 4 | 1 | 5 | 34 |
| Zambia | Community health worker | 90 | 0 | 1 | 0 | 1 | 91 |
| Zambia | Community health worker | 32 | 0 | 1 | 1 | 2 | 34 |
| Zambia | Community health worker | 6 | 0 | 0 | 0 | 0 | 6 |
| Zambia | Community health worker | 2 | 0 | 0 | 0 | 0 | 2 |
| Zambia | Community health worker | 13 | 0 | 1 | 0 | 1 | 14 |
| Zambia | Env. health technician | 326 | 3 | 17 | 4 | 24 | 350 |
| Zambia | Lab technician | 84 | 0 | 2 | 0 | 2 | 86 |
| Zambia | Lab technician | 81 | 0 | 0 | 0 | 0 | 81 |
| Zambia | Lab technician | 60 | 0 | 0 | 0 | 0 | 60 |
| Zambia | Nurse | 154 | 0 | 8 | 0 | 8 | 162 |
| Zambia | Nurse | 55 | 0 | 2 | 0 | 2 | 57 |
| Zambia | Nurse | 133 | 0 | 2 | 0 | 2 | 135 |
| Zambia sub total | | **1197** | **3** | **43** | **11** | **57** | **1254** |
| Tanzania | Nurse | 15 | 0 | 0 | 1 | 1 | 16 |
| Tanzania | Nurse | 84 | 0 | 0 | 0 | 0 | 84 |
| Tanzania | Nurse | 115 | 0 | 0 | 0 | 0 | 115 |
| Tanzania | Nurse | 218 | 0 | 2 | 0 | 2 | 220 |
| Tanzania | Nurse | 239 | 0 | 1 | 0 | 1 | 240 |
| Tanzania | Nurse | 97 | 0 | 1 | 0 | 1 | 98 |
| Tanzania | Nurse | 20 | 0 | 1 | 1 | 2 | 22 |
| Tanzania | Nurse | 73 | 0 | 1 | 0 | 1 | 74 |
| Tanzania | Nurse | 149 | 0 | 1 | 0 | 1 | 150 |
| Tanzania | Nurse | 177 | 0 | 1 | 2 | 3 | 180 |
| Tanzania | Nurse | 72 | 0 | 0 | 0 | 0 | 72 |
| Tanzania | Nurse | 348 | 0 | 4 | 0 | 4 | 352 |
| Tanzania | Nurse | 184 | 1 | 2 | 0 | 3 | 187 |
| Tanzania | Nurse | 258 | 0 | 0 | 0 | 0 | 258 |
| Tanzania | Nurse | 66 | 0 | 0 | 0 | 0 | 66 |
| Tanzania | Nurse | 94 | 0 | 0 | 0 | 0 | 94 |
| Tanzania | Nurse | 149 | 1 | 0 | 0 | 1 | 150 |
| Tanzania | Nurse | 231 | 0 | 1 | 1 | 2 | 233 |
| Tanzania | Nurse | 214 | 0 | 1 | 0 | 1 | 215 |
| Tanzania sub total | | **2803** | **2** | **16** | **5** | **23** | **2826** |
| Grand total | | **4000** | **5** | **59** | **16** | **80** | **4080** |
